# Controlled Experiments Reveal Moderate, Nonlinear Relationships Between eDNA Concentration and Fish Biomass in Three Freshwater Species of Monitoring Relevance

**DOI:** 10.1002/ece3.73129

**Published:** 2026-02-17

**Authors:** Lorenzo Talarico, Gerardo Petrosino, Anna Rita Rossi, Paolo Franchini, Lorenzo Tancioni

**Affiliations:** ^1^ Laboratory of Experimental Ecology and Aquaculture, Department of Biology University of Rome Tor Vergata Rome Italy; ^2^ Department of Ecology and Biology (DEB) Tuscia University Viterbo Italy; ^3^ Department of Biology and Biotechnology “C. Darwin” Sapienza University of Rome Rome Italy

**Keywords:** eDNA filtering, endemic species, environmental DNA, generalized additive mixed models, invasive species, quantitative PCR

## Abstract

Understanding the relationship between environmental DNA (eDNA) concentration and taxa abundance is essential for the advancement of quantitative biodiversity monitoring. We experimentally manipulated biomass of three freshwater fish species of monitoring interest—the Italian‐endemic *Squalius lucumonis* and the exotic‐invasive *Pseudorasbora parva* and *Lepomis gibbosus* —under controlled conditions (flow‐through 310 and 1330 L tanks). Following eDNA collection (2 L water filtration) and Real‐Time PCR quantification, Generalized Additive Mixed Models (GAMMs) revealed: (i) monotonic non‐linear relationships of moderate‐to‐high magnitude (0.42 < partial‐*R* ^2^ < 0.62), with eDNA concentrations plateauing at intermediate biomasses in smaller‐sized taxa; and (ii) a significant effect of experimental replicates (tanks) in two out of three species. These findings suggest that eDNA‐based biomass quantification should not assume linearity, and emphasize the critical role of replication to account for inherent uncertainty, likely driven by inter‐ and intra‐individual variations in eDNA shedding rates.

## Graphical abstract

Environmental DNA (eDNA) is a sensitive and non‐invasive tool to assist biodiversity monitoring. However, quantitative monitoring is limited by the often uncertain association between eDNA amount and fish abundance. Here we assessed the relationship between eDNA concentration (quantified by qPCR) and biomass of three freshwater fish of monitoring interest (*Squalius lucumonis*, *Lepomis gibbosus* , and *Pseudorasbora parva* ) in controlled conditions, revealing moderate and non‐linear relationships, with plateauing curves in two out of the three examined species. Replicates' variability also emerged as a source of uncertainty. This contributes to improving eDNA‐based monitoring and understanding patterns of eDNA shedding.

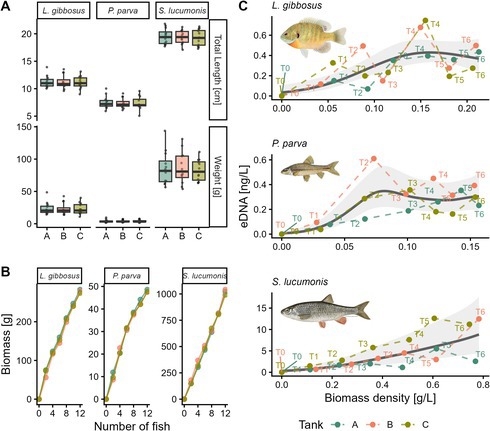

## 1 Introduction

Environmental DNA (eDNA) refers to fragments of DNA that organisms release into environmental matrices through skin, feces, excretions or dead cells, which can then be used to detect “source” taxa by DNA‐barcoding/metabarcoding (Taberlet et al. 2018). eDNA sampling is rapid, noninvasive and cost‐effective, and often provides reliable and sensitive results, so that it is considered a valuable complementary tool to improve biomonitoring of either native threatened species or invasive ones, especially in freshwater ecosystems (Rees et al. 2014; Rourke et al. 2022; Takahashi et al. 2023). Among target taxa, fishes have received particular attention, likely because they are important bioindicators (Pont et al. 2021) and/or include species of commercial/conservation value, such as cyprinds and salmonids (Rourke et al. 2022). Importantly, 90% of species‐specific studies found a positive relationship between eDNA concentrations and measures/estimates of fish abundance, either in natural or controlled conditions. However, the understanding of how eDNA concentrations relate to fish density remains largely incomplete. Mutually interacting biotic and abiotic factors can influence this relationship by determining, among others, rates of eDNA sheeding, such as intraspecific variation related to metabolism and fish size, or differences in water flow, temperature, and depth (reviewed in Rourke et al. 2022). Consequently, the relationship between eDNA concentration and fish biomass should be assessed in controlled experiments to minimize confounding effects, ideally as a first step before validating results in natural ecosystems (e.g., Takahara et al. 2012).

Here, we targeted three freshwater species occurring in Italian freshwaters—the central Italy endemic brook chub (*Squalius lucumonis*, hereafter *Sl*), the North American native pumpkinseed ( *Lepomis gibbosus* , hereafter *Lp*), and the East Asian native topmouth gudgeon ( *Pseudorasbora parva* , hereafter *Pp*)—whose monitoring is essential for opposite reasons. While the former is listed as “critically endangered” by the International Union for Conservation of Nature (IUCN) and thus worthy of special conservation effort, the latter two are listed as invasive alien species of European Union concern, and their management is foreseen by the EU Regulation no. 1143/2014 (https://eur‐lex.europa.eu/eli/reg/2014/1143/oj/eng).

Several biomonitoring‐oriented studies have successfully used eDNA to infer the occurrence of *Pp* and *Lg* in natural and/or artificial (controlled) environments (e.g., Keskin 2014; Davison et al. 2016; Manfrin et al. 2022; Davison and Copp 2023; Mirone 2024), while only a single study tested the in situ detection of *Sl* (Mirone 2024). However, to our knowledge, no study has directly investigated the relationship between eDNA concentration and their biomass to date. To address this gap, we conducted experiments under controlled conditions (non‐recycled flowing water in medium or large volume tanks), systematically varying fish biomass to simulate different fish abundances in natural lentic or slow‐flow mesohabitats where fish aggregate, such as ponds or pools in small watercourses, and measured eDNA amount in water samples. Outcomes from this study will constitute a necessary step to move from qualitative to quantitative monitoring of these taxa, while contributing to understanding the association pattern between eDNA amount and fish abundance.

## 2 Materials and Methods

### 2.1 Experimental Design

**FIGURE 1:**
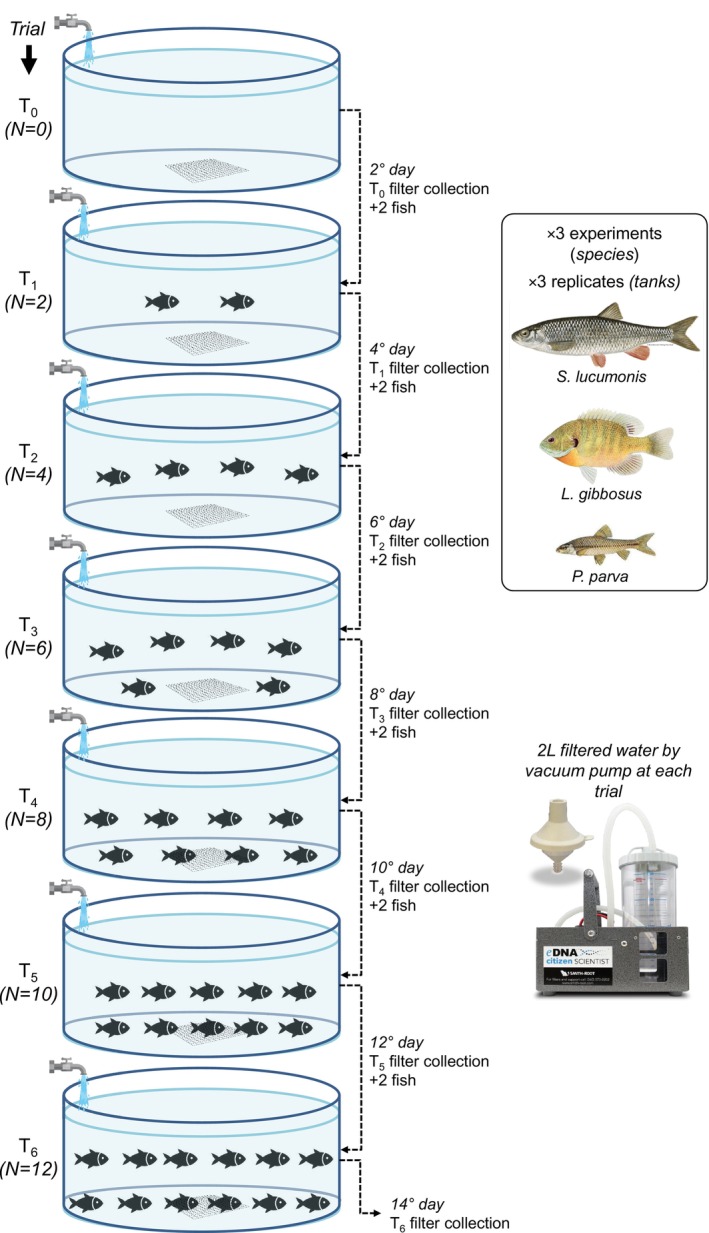
Graphical schematic of the experimental design for eDNA collection in controlled conditions at increasing fish biomasses for three target species.

**FIGURE 2:**
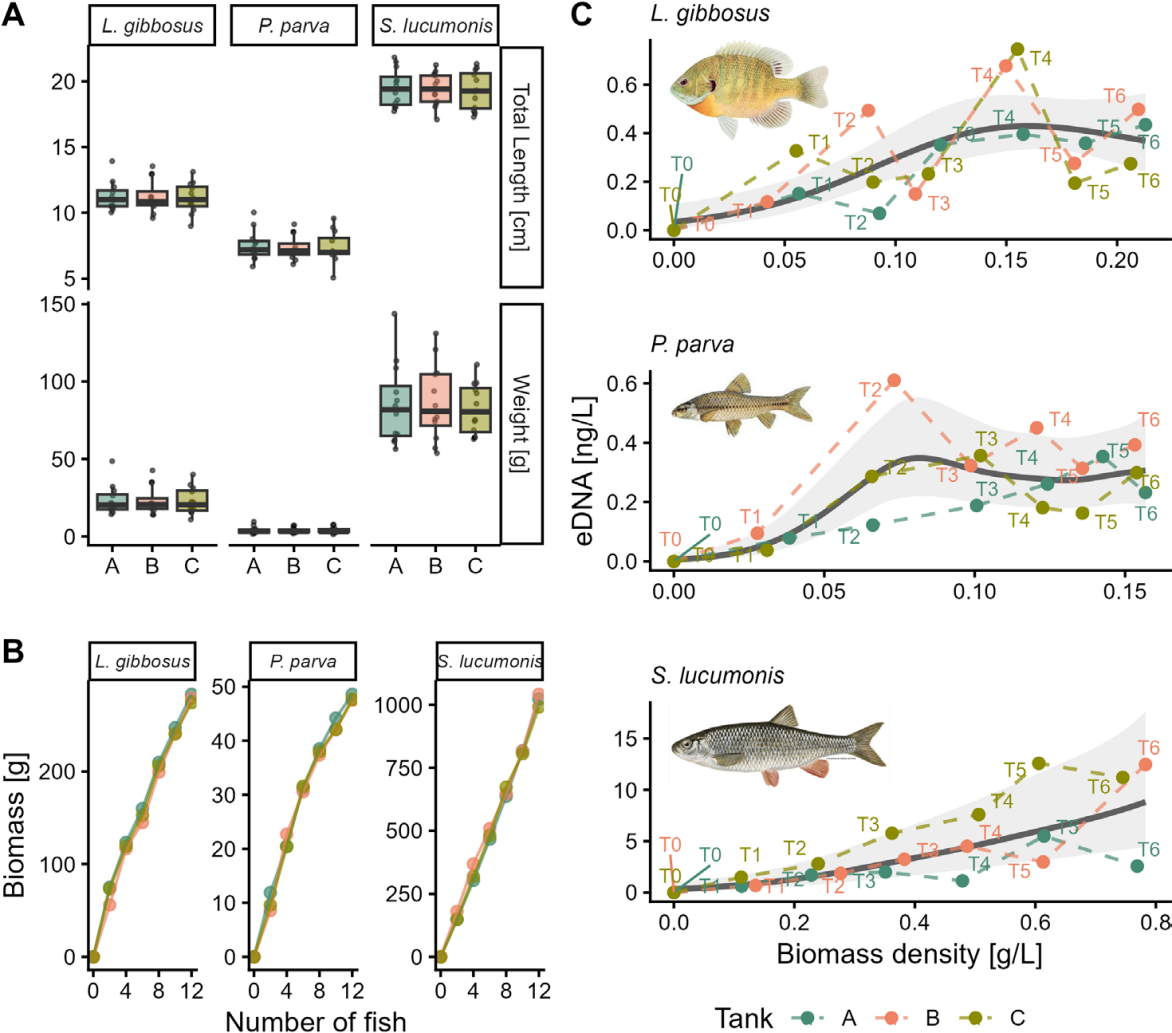
For each of three target species and experimental tanks (replicates): (A) the distribution (boxplots) of fish sizes; (B) the variation in cumulative biomass at increasing fish number; (C) the fit of GAMMs depicting the average smooth effect of fish biomass density on eDNA concentration for the three target species, along with the variation of estimated eDNA concentration across trials (from T_0_ to T_6_). Model predictions and confidence intervals (gray‐shaded area) were generated using the *predict* R function (excluding random effect), with eDNA concentrations back‐transformed using the exponential function to improve model interpretability.

The experimental design, the same for the three target species, consisted of seven trials (Figure 1)—a “blank” with no fish (T_0_), followed by six trials with increasing fish abundance (from T_1_ = 2 fish, to T_6_ = 12 fish) obtained by moving two fish of similar size from a stocking tank into the experimental tank every 48 h—performed in triplicate, namely in three independent circular tanks. Tank volumes were 1330 L for *Sl* and *Lg*, and 310 L for *Pp*. Such a volume difference was necessary to optimize the biomass‐to‐water volume ratio, compensating for the smaller *Pp* body mass (Figure 2A) while preventing excessive eDNA dilution below detection limits. Tanks were supplied with a constant inlet of well water (thus free of any fish DNA contamination), ensuring approximately 15 and 36 complete water renewals per day, respectively—notably, such a rapid water turnover (a complete renewal ~40–95 min) would minimize possible confounding effects due to eDNA decay or long‐time accumulation within tanks. Experimental tanks were equipped with aerators, and water was discharged through a fine mesh grid at the bottom. Experiments were conducted in the indoor hatchery of the Laboratory of Experimental Ecology and Aquaculture (University of Rome “Tor Vergata”) with an artificial 10‐h photoperiod; however, due to structural and logistic constraints, they occurred in different months of 2024 (Table S1). The fish used in this study were already available in mesocosms and indoor tanks of the above‐mentioned laboratory.

### 2.2 Monitoring of Experimental Conditions, eDNA Collection and Fish Measures

Every 48 h, we collected eDNA by filtering 2 L of water from each tank using the vacuum pump with 0.45 μm sterile filters of the eDNA Citizen Scientist Kit (Smith‐Root; https://www.smith‐root.com/edna/edna‐citizen‐scientist‐sampler), and stored filters at −20°C in separate plastic bags until eDNA extraction. Then, we released two fish into each experimental tank. To ensure approximately similar biomasses among the three replicates, we used fish of comparable size from a stocking tank and annotated their individual total length (TL) and weight (W). We verified the homogeneity of experimental conditions by measuring temperature, pH, and dissolved oxygen in each tank with a Hanna HI98194 multiparametric probe.

To reduce DNA contamination, the tanks' surfaces were cleaned with pure bleach just before starting experiments. Operators wore disposable nitrile gloves and face masks during operations, and equipment was sterilized by soaking for approximately 2 h in pure bleach at the end of each trial session. For the duration of the experiments, fish in the trial tanks were not fed (Davison et al. 2016; Karlsson et al. 2022) to avoid possible biases in eDNA shedding associated with different individual feeding and metabolic activity (reviewed in Rourke et al. 2022).

### 2.3 DNA Extraction, qPCR and Calibration

eDNA extractions were carried out in a dedicated laboratory, whose surfaces were cleansed with 10% bleach before any operation. We followed the manufacturer's protocol of the Quick‐DNA Miniprep kit (Zymo Research) to extract eDNA from the (half) encapsulated filtering membrane, eluting eDNA in a final 60 μL volume of the elution buffer. We obtained a total of 66 samples: seven extractions from trials (including the T_0_ blank) ×3 replicates (tanks) and ×3 experiments (target species), plus 3 extraction blanks (one per species) to check for contamination of reagents.

qPCRs were performed on a CFX Opus 96 Real‐Time PCR System (BioRad), using primers and probes targeting mitochondrial fragments (see details in Table S2). The reaction mixture consisted of: 1× GoTaq Probe qPCR Master Mix (Promega), 0.5 μM each primer, 0.25 μM specific TaqMan probe, 2 μL of the eDNA extraction sample, and PCR‐grade water in a final volume of 20 μL. The thermal cycling profile was the same for all three species: 98°C for 3′, 50 cycles at 95°C for 10″ and species‐specific annealing temperature (Table S2) for 30″. Each sample was qPCR amplified in triplicate, and the quantification cycle (Cq) was calculated as the mean of the three measures. Negative qPCR controls (only reagents) were also analyzed in triplicate for each run. To estimate eDNA concentration (ng/μL), we created the standard curve for each species, through triplicate amplification of five serial dilutions (from 10^−1^ to 10^−5^) obtained from two samples of tissue‐derived genomic DNA (range 22–58 ng/μL). The eDNA concentration was finally expressed as ng/L.

### 2.4 Statistical Analyses

Firstly, we used the *aov* function in R (R Core Team 2024) to perform a nested analysis of variance (ANOVA) to test for differences in fish size (TL and W, separately) between replicates and species: we expected an obvious difference between species but not among replicates since we employed fish of similar size.

For each species, we assessed the relationship between eDNA concentration and fish biomass density while accounting for potential tank‐specific effects. We employed the *gam* function in the “mgcv” v.1.9‐3 R‐package (Wood 2011) to fit Generalized Additive Mixed Models (GAMMs). These models used the Restricted Maximum Likelihood (REML) algorithm and a Tweedie error distribution (suitable for continuous non‐negative data with potential overdispersion) with a logarithmic link function. The model structure was defined as follows:

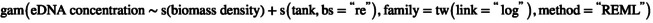

where “eDNA concentration” [ng/L] is the response variable, “biomass density” [g/L] is the smoothed fixed predictor, and “tank”’ is included as a random effect to account for the non‐independence of repeated measures within the same experimental unit. The significance of smooth terms and random effects was assessed via *F*‐tests. To quantify the relative importance of each component, we performed hierarchical partitioning of variance using the *gam.hp* function of the homonymous R package (Lai et al. 2024), calculating the individual (partial) contribution of predictors to the overall adjusted‐*R* ^2^.

## 3 Results

Water parameters were approximately constant during experiments (Table S1), and fish were of comparable sizes among replicates within species (nested‐ANOVA: *F* = 0.06 and *p* = 0.999 for TL; *F* = 0.09 and *p* = 0.997 for W; Figure 2A and Table S1)—but obviously they were not across species (nested‐ANOVA: *F* = 908.4 and *p* < 0.0001 for TL; *F* = 316.3 and *p* < 0.0001 for W)—guaranteeing an inter‐replicate homogeneous increase in cumulative biomasses at increasing fish densities (Figure 2B).

Overall, Cq values for eDNA samples ranged between 25.4 and 35.2 (Supporting Information). Tissue‐derived qPCR standard curves yielded the following species‐specific linear regressions and efficiency parameters: *y* = −4.195*x* + 23.414 (*R* ^2^ = 0.977, *E* = 73.1%) for *Lg*; *y* = −3.990*x* + 23.197 (*R* ^2^ = 0.994, *E* = 78.1%) for *Pp*; and *y* = −4.102*x* + 24.341 (*R* ^2^ = 0.984, *E* = 75.3%) for *Sl*. For *Sl*, the calibration curve was established using four serial dilutions, as the 10^−5^ dilution fell below the limit of detection. Despite the observed suboptimal efficiencies, high *R* ^2^ values confirmed strong internal consistency for eDNA quantification across the detected range. Across trials, eDNA concentration was sharply higher in *Sl* (range 0.63–12.60 ng/L) than in *Lg* (range 0.07–0.75 ng/L) and *Pp* (range 0.04–0.61 ng/L). Controls did not return signals of DNA, indicating no contamination in the experimental tanks at the beginning of the experiments (T_0_ blanks) or in the reagents (Figure 2C).

According to GAMMs, fish biomass was a significant predictor of eDNA concentration for all species, with partial adjusted‐*R* ^2^ values ranging from 0.42 (*Lg*) to 0.62 (*Pp*; Table 1). The fitted curves indicated an initial eDNA increase with increasing fish biomass, followed by a rough plateau at higher biomass densities for *Pp* and *Lg*. Conversely, a positive, non‐linear relationship emerged for *Sl* (Figure 2C). The individual tanks significantly contributed to the variance explained by GAMMs for *Sl* and *Pp*, but not for *Lg* (Table 1).

**TABLE 1:** Results of the fitted GAMMs (family = Tweedie, link = Log) assessing the relationship between qPCR‐based estimates of eDNA concentration (ng/L) and smoothed fish biomass density, while controlling for non‐independence of trials within the experimental tank (smoothed random effect).

Species	Predictor	Edf	*F*	*p*	Partial adjusted‐*R* ^2^	Overall adjusted‐*R* ^2^
*Lepomis gibbosus*	Biomass density (smooth term)	1.89	8.39	**0.003**	0.420 (100%)	0.420
	Tank (random effect)	< 0.01	0.00	0.601	0.000 (0.0%)	
*Pseudorasbora parva*	Biomass density (smooth term)	3.97	6.96	**0.002**	0.623 (84.6%)	0.736
	Tank (random effect)	1.62	4.61	**0.015**	0.113 (15.4%)	
*Squalius lucumonis*	Biomass density (smooth term)	2.40	17.95	**< 0.001**	0.613 (76.1%)	0.805
	Tank (random effect)	1.81	9.76	**0.001**	0.193 (23.9%)	

*Note:* For each predictor, the following information is provided: Effective degree of freedom (Edf), *F*‐statistic and associated *p*‐value (statistically significant *p*‐values are in bold), and relative contribution (partial adjusted‐*R* ^2^ and %) to the overall variance explained by the model (overall adjusted‐*R* ^2^). Further details on models are shown in Table S3.

## 4 Discussion

We investigated the relationship between eDNA concentration and cumulative biomass density in three fish species of conservation and management interest, under controlled conditions roughly simulating lentic or slow‐flowing freshwater (meso)habitats. At first, target eDNA was successfully detected at the lowest density trial (T_1_; biomass range: 0.03–0.14 g/L) across all species and replicates, 48 h after fish introduction. This is particularly remarkable given the substantial tank volumes and the high water renewal rate of our flow‐through system. While these results represent the first eDNA records for *S. lucumonis*, Davison et al. (2016) detected eDNA for the other two species at comparable or lower biomass densities 24 h after stocking. However, those findings were obtained in closed experimental systems—specifically, 0.02 g/L for both *P. parva* and *L. gibbosus* in 44 L static aquaria, and 0.003 g/L for *L. gibbosus* in outdoor mesocosms with recirculating water—further highlighting the efficiency of our detection even under high turnover conditions.

Secondly, we found moderate relationships between eDNA concentration and biomass (partial adjusted‐*R* ^2^ = 0.42–0.62) in the tested taxa for the first time, adding evidence to a generally established pattern for fish, although to a varying and often incomparable extent—indeed, comparing prediction power remains challenging because experimental set‐up and statistical approaches differ across studies. Merely by way of example, Karlsson et al. (2022) obtained robust linear relationships between eDNA concentration and pike biomass at different time points for juveniles in aquaria (*R* ^2^ = 0.87) and adults in mesocosms (*R* ^2^ = 0.74). Analogously, common carp biomass was linearly linked to eDNA concentration (*R* ^2^ = 0.66) in 9 L aquaria experiments with no water renewal (Takahara et al. 2012). Benoit et al. (2023) found a “global” moderate linear relationship (*R* ^2^ = 0.33) between eDNA concentration and biomass of juvenile Pacific salmon in mesocosms, although the strength (and slope) of relationships markedly varied across experiment timepoints (0.16 < *R* ^2^ < 0.90).

Interestingly, and in contrast to previously mentioned studies, we observed non‐linear relationships across all examined taxa. The effective degrees of freedom (edf), ranging from 1.89 to 3.97 (Table 1), indicate a moderate‐to‐strong degree of non‐linearity in the eDNA‐biomass associations (Zuur et al. 2009). This was particularly evident for *Pp* and *Lg*, where eDNA concentrations plateaued at intermediate fish abundances approximately 6–8 days after the start of stocking (corresponding to trials T_3_–T_4_), as opposed to *Sl* that showed a monotonic nonlinear relationship. We hypothesize that at intermediate densities, the eDNA released by fish reached a dynamic equilibrium with the eDNA lost through the water outlet (Benoit et al. 2023). In our setup, eDNA decay (estimated at 1.5%–15.9% hourly reduction across various species; Maruyama et al. 2014 and citations therein) and long‐term accumulation were likely negligible, as the water residence time (40–96 min) was sufficiently short to prevent any significant degradation from occurring. Consequently, for smaller‐sized taxa (*Pp* and *Lg*), the system reached a steady state within the tested biomass range. In contrast, since the biomass of *Sl* was fourfold greater than that of the other two species, the rate of eDNA production likely consistently exceeded the discharge rate, preventing the attainment of a plateau. Beyond mere biomass scaling, we cannot exclude that physiological factors associated with larger body sizes might have played a role. It is possible that the higher biomass density of *Sl* induced a subtle physiological response to confinement, potentially increasing eDNA shedding rates through enhanced mucus production or metabolic excretion (Pilliod et al. 2014; Klymus et al. 2015). Regardless of the underlying mechanism, these findings suggest that the attainment of a dynamic equilibrium is highly dependent on the specific interaction between species' biomass and the system's hydrological turnover.

Similar non‐linear patterns between eDNA concentration and biomass have been verified observed in natural ecosystems, albeit in a limited number of studies. For instance, Coulter et al. (2019) demonstrated eDNA levels plateauing at intermediate densities of Silver Carp ( *Hypophthalmichthys molitrix* ) across a 460 km river transect, while a non‐linear relationship (reaching saturation only in part) was found between Ayu Sweetfish ( *Plecoglossus altivelis* ) abundance/biomass and eDNA in small Japanese streams (Doi et al. 2017). Notably, such relationships are often “linearized” through logarithmic transformations for practical modeling purposes (e.g., Klymus et al. 2015; Rourke et al. 2023), a procedure that may mask underlying non‐linear patterns. Further investigations using appropriate statistical frameworks are needed to clarify whether non‐linear models better approximate eDNA dynamics in natural inland waters with moderate flow.

Finally, we found a non‐negligible effect of replicates in two out of three experiments. Since our experimental setup minimized major known confounding factors—such as variations in individual size, life stage, feeding activity, water temperature, and flow (Rourke et al. 2022)—this variance could be due to non‐independence of sequential biomass measures within the same tank. This underscores the impact of even minimal differences in individual biomass among replicates (Figure 2B). Additionally, we cannot rule out the influence of subtle concomitant factors affecting inter‐ and intra‐individual eDNA shedding rates, such as metabolic rate, motility, or stress (Klymus et al. 2015; Rourke et al. 2023).

In conclusion, our findings corroborate that eDNA is an effective, non‐invasive tool for the quantitative monitoring of freshwater fish, particularly for the taxa examined in this study. Future validation efforts in natural systems should include multiple biological and technical replicates to better account for the inherent uncertainty of the eDNA‐biomass relationships, whose linearity should not be assumed. Furthermore, moving toward more complex, multi‐species (experimental) communities is crucial to clarify how interspecific interactions and source abundance affect eDNA dynamics under more realistic environmental conditions.

## Author Contributions

**Lorenzo Talarico:** conceptualization (equal), formal analysis (lead), investigation (equal), visualization (lead), writing – original draft (lead), writing – review and editing (equal). **Gerardo Petrosino:** investigation (equal), writing – original draft (supporting), writing – review and editing (equal). **Anna Rita Rossi:** conceptualization (equal), funding acquisition (equal), resources (equal), writing – review and editing (equal). **Paolo Franchini:** funding acquisition (equal), resources (equal), writing – review and editing (equal). **Lorenzo Tancioni:** conceptualization (equal), funding acquisition (equal), investigation (equal), resources (equal), supervision (equal), writing – review and editing (equal).

## Funding

This study was funded by the “Italian Ministry of University and Research” through the PRIN 2022‐2022JJ7STN project.

## Ethics Statement

Fishes used in this study were already confined in mesocosms and indoor tanks of the Laboratory of Experimental Ecology and Aquaculture of the Tor Vergata University (Rome, Italy). No specific ethical approval was required, as the study did not involve any impactful handling or sacrifice of specimens.

## Conflicts of Interest

The authors declare no conflicts of interest.

## Supporting information


**Tables S1–S3:** ece373129‐sup‐0001‐DataS1.docx.

**Data S1:** ece373129‐sup‐0002‐DataS2.csv.

## Data Availability

Raw data are provided in the Supporting Information.
