# Supplementary material for: Controlled Experiments Reveal Moderate, Nonlinear Relationships Between eDNA Concentration and Fish Biomass in Three Freshwater Species of Monitoring Relevance
Source: Ecol Evol. 2026 Feb 17;16(2):e73129. doi: 10.1002/ece3.73129 (PMC12912921; doi:10.1002/ece3.73129)
Supplement: Supplementary file 1 — Tables S1–S3: ece373129‐sup‐0001‐DataS1.docx. [file ECE3-16-e73129-s002.docx]

**Supplementary Information**

“Controlled experiments reveal moderate, nonlinear relationships between eDNA concentration and fish biomass in three freshwater species of monitoring relevance”

*L. Talarico, G. Petrosino, A.R. Rossi, P. Franchini, L. Tancioni*

***Table S1***

Summary information on fish metrics and experimental conditions for three experiments (Species) and three replicates (Rep) across all trials: the period of experiment (Month); the cumulative weight of fish at the end of experiments (W tot); the average per-fish weight (W mean) and total length (TL mean); the tank volume (Volume); and the inlet flow (Flow), temperature (Temp), pH (pH) and dissolved oxygen (DO) of water. Standard deviations are provided in parentheses for averaged values.

| **Species** | **Rep** | **Month** | **W tot [g]** | **W mean [g]** | **TL mean [cm]** | **Volume [L]** | **Flow [L/s]** | **Temp [°C]** | **pH [units]** | **DO [mg/L]** |
| --- | --- | --- | --- | --- | --- | --- | --- | --- | --- | --- |
| *S .lucumonis* | A | February | 1022.9 | 85.2 (26.2) | 19.5 (1.4) | 1330 | 0.23 (0.01) | 18.02 (0.10) | 6.58 (0.08) | 7.38 (0.25) |
|  | B | February | 1041.1 | 86.8 (24.7) | 19.4 (1.2) | 1330 | 0.23 (0.03) | 18.04 (0.12) | 6.54 (0.10) | 7.27 (0.18) |
|  | C | February | 991.0 | 82.6 (16.5) | 19.3 (1.4) | 1330 | 0.22 (0.01) | 18.04 (0.11) | 6.60 (0.17) | 7.35 (0.21) |
| *P. parva* | A | September | 48.6 | 4.1 (2.3) | 7.5 (1.1) | 310 | 0.13 (0.02) | 18.06 (0.21) | 6.91 (0.06) | 9.52 (0.07) |
|  | B | September | 47.5 | 4.0 (1.7) | 7.3 (0.9) | 310 | 0.13 (0.02) | 18.06 (0.20) | 7.00 (0.05) | 9.53 (0.06) |
|  | C | September | 47.7 | 4.0 (1.9) | 7.4 (1.2) | 310 | 0.12 (0.02) | 18.09 (0.22) | 7.03 (0.09) | 9.62 (0.08) |
| *L. gibbosus* | A | November | 283.0 | 23.6 (9.6) | 11.2 (1.1) | 1330 | 0.24 (0.01) | 17.84 (0.24) | 6.59 (0.20) | 9.12 (0.17) |
|  | B | November | 278.8 | 23.2 (9.1) | 11.2 (1.2) | 1330 | 0.24 (0.01) | 17.87 (0.26) | 6.57 (0.08) | 8.91 (0.09) |
|  | C | November | 274.1 | 22.8 (8.7) | 11.1 (1.2) | 1330 | 0.24 (0.01) | 17.78 (0.29) | 6.61 (0.06) | 9.07 (0.15) |

***Table S2***

Sequences and reference of primers/probes used for qPCRs in the three examined species, along with information on target mitochondrial DNA fragments and the annealing temperature of the qPCR cycling step. All probes were marked with a fluorescent 5’-FAM dye, and a 3’-Black Hole Quencher® -1 (BHQ-1) quencher.

| **Species** | **mtDNA gene** | **Fragment length [bp]** | **5’-primer forward-3’** | **5’-primer reverse-3’** | **5’-Probe-3’** | **Annealing temperature [°C]** | **Reference** |
| --- | --- | --- | --- | --- | --- | --- | --- |
| *S .lucumonis* | Cytochrome-b (CYT-B) | 153 | GCATTGTGAAACTTCGGTTCC | GGTGACGGATGAAAACGC | CCTGGCAATGCACTACACTTCCG | 58 | Mirone, 2024 ^(1)^ |
| *P. parva* | Cytochrome Oxidase I (COI) | 79 | CCCTGACATAGCATTCCCC | AGAGGCTAAGAGTAGGAGGA | AAGGGGGAAGTAGTCAGAAGCT | 64 | Manfrin et al., 2022 ^23^ |
| *L. gibbosus* | Cytochrome Oxidase I (COI) | 83 | GCTGGCACGGGCTGAA | GAGAAAATAGTGAGATCAACGGATGCT | CCGGCAACCTAGCCCACGCC | 60 | Davison & Copp, 2023 ^(3)^ |

^(1)^ [Mirone, E. (2024). Non-invasive monitoring of freshwater species in Central and Southern Italian basins. From standard survey to environmental DNA. PhD thesis, University of Molise, Campobasso (Italy).](https://www.zotero.org/google-docs/?8NvTwx)

^(2)^ Manfrin, C., Mirimin, L., Zanetti, M., Pizzul, E., Giulianini, P.G. & Pallavicini, A. (2022). Highly sensitive environmental DNA detection of topmouth gudgeon, *Pseudorasbora parva*: a comparison of qPCR and microfluidic qdPCR. *Biological Invasions*, 24(7), 2121–2133. https://doi.org/10.1007/s10530-022-02761-2

^(3)^ Davison, P.I. & Copp, G.H. (2023). A rapid assessment of non-native fish distributions in two English river basins using environmental DNA. *Journal of Vertebrate Biology*, 72(22068). https://doi.org/10.25225/jvb.22068

***Table S3***

Additional information on the parameters of fitted GAMMs

| **Species (model)** | **REML** | **Tweedie power parameter** | **Intercept** | | | |
| --- | --- | --- | --- | --- | --- | --- |
|  |  |  | **estimate** | **standard error** | **t value** | **p-value** |
| *L. gibbosus* | -5.661 | 1.01 | -1.513 | 0.148 | -10.23 | **<0.001** |
| *P. parva* | -11.793 | 1.09 | -2.004 | 0.222 | -9.015 | **<0.001** |
| *S. lucumonis* | 37.636 | 1.02 | 0.809 | 0.343 | 2.36 | **0.031** |
